# Unmanned Aerial Vehicle-Assisted Terahertz–Visible Light Communication Systems: An In-Depth Performance Analysis

**DOI:** 10.3390/s24134080

**Published:** 2024-06-23

**Authors:** Hanojhan Rajahrajasingh, Dushantha Nalin K. Jayakody

**Affiliations:** 1 COPELABS, Lusofona University, 1749-024 Lisbon, Portugal; hanojhansingh@gmail.com; 2 Department of Computer Systems Engineering, Faculty of Computing, Sri Lanka Institute of Information Technology, Malabe 10115, Sri Lanka; 3 Centre of Excellence in Informatics, Electronics and Transmission (CIET), Faculty of Engineering, Sri Lanka Institute of Information Technology, Malabe 10115, Sri Lanka

**Keywords:** BER, outage probability, THz communications, VLC, UAV

## Abstract

This paper investigates the performance of dual-hop unmanned aerial vehicle (UAV)-assisted communication channels, employing a decode-and-forward (DF) relay architecture. The system leverages terahertz (THz) communication for the primary hop and visible light communication (VLC) for the secondary hop. We conduct an in-depth analysis by deriving closed-form expressions for the end-to-end (E2E) bit error rate (BER). Additionally, we use a Monte Carlo simulation approach to generate best-fitting curves, validating our analytical expressions. A performance evaluation through BER and outage probability metrics demonstrates the effectiveness of the proposed system. Specifically, our results indicate that the proposed system outperforms Free-Space Optics (FSO)-VLC and Radio-Frequency (RF)-VLC at a higher signal-to-noise ratio (SNR). The results of this study provide valuable insights into the feasibility and limitations of UAV-assisted THz-VLC communication systems.

## 1. Introduction

Wireless communication systems have seen significant transformations since their origin. Today, advanced technologies such as autonomous vehicles, smart devices, and the Internet of Things (IoT) are ubiquitous, all hinging on wireless communication to meet the growing demand for high bandwidth and fast data rates. Over the years, mobile communication has progressed from the first generation (1G) through successive iterations—2G, 3G, 4G, and now 5G. Additionally, Wi-Fi standards (IEEE 802.11) for short-range wireless communication have rapidly advanced, particularly in terms of capacity, reliability, data rate, and medium access methods, in order to meet the needs brought about by the growing demand of data-hungry applications over the past decades, which follows the Edholm’s law; this law states that the data rate and bandwidth will double every 1.5 years. Following a similar trend, total mobile network traffic is forecast to reach approximately 563 EB per month by the end of 2029. The predicted traffic increase until 2029 assumes that the early adoption of Extended Reality (XR)-type services, such as Augmented Reality (AR), Virtual Reality (VR), and Mixed Reality (MR), will occur in the latter part of the projection period [1]. This trend is causing a radio frequency crunch and forcing the research community to explore ways to acquire larger bandwidth and higher data rates. THz communications and VLC have the potential to be the feeders for this growing demand for data rates and bandwidth [2].

VLC is emerging as a promising technology to complement traditional RF communication systems, addressing the increasing demand for higher data rates and broader bandwidth. VLC utilizes the visible spectrum (400–700 nm), leveraging light-emitting diodes (LEDs) for dual purposes: illumination and high-speed data transmission. This dual functionality makes VLC an attractive solution for environments where RF communication is challenging or restricted, such as in densely populated urban areas, hospitals, and underwater environments [3]. Furthermore, VLC offers several advantages, including immunity to electromagnetic interference, enhanced security due to the line-of-sight (LoS) nature of light propagation, and the vast, unlicensed visible spectrum, which alleviates the spectrum scarcity issue associated with RF communication [4].

THz communication is another emerging frontier in wireless technology that operates in the frequency range between 0.1 THz and 10 THz. THz communication exploits the untapped spectrum between microwave and infrared frequencies. This enables ultra-high-speed data transmission, which is essential for next-generation applications such as ultra-high-definition video streaming, real-time VR, and advanced scientific imaging. THz waves offer remarkable advantages, including the ability to carry vast amounts of data and support extremely high bandwidth, which is crucial for mitigating the radio frequency spectrum crunch. However, THz communication also presents challenges, such as a limited transmission range and significant atmospheric attenuation. To overcome these obstacles, research is focused on developing advanced materials, innovative antenna designs, sophisticated signal processing techniques, and the use of mobile base stations [5,6].

Due to the limitations of VLC and THz communications, these technologies are often combined with other communication technolgies such as RF, FSO, or Power Line Communication (PLC) through the use of suitable relay architectures. Many studies have integrated UAVs into these relay architectures, leveraging their ability to function as mobile base stations and adjust their positions to enhance network reliability and coverage [7,8].

Several studies have been conducted on the interoperability of different communication technologies using DF relay architecture. In terms of VLC, this is often combined with RF, FSO, or PLC [7,9,10]. In [4], the authors conduct an examination of hybrid RF/VLC systems, emphasizing the integration of RF and VLC technologies. The study explores various network topologies, performance analyses, and system-level simulations of hybrid RF/VLC systems, showcasing their potential benefits in terms of connectivity, throughput, coverage, and energy efficiency. By discussing research directions and emerging applications, the authors highlight the significance of hybrid RF/VLC systems in improving user rates, mobility, network capacity, and overall network optimization. In [11], the researchers developed an indoor hybrid PLC/VLC/infrared (IR) communication system based on the TCP/IP model. The system integrates PLC, VLC, and IR to enable full-duplex communication between two personal computers. By utilizing a virtual network interface and implementing Carrier-Sense Multiple Access (CSMA) as a medium access technique, the system achieves a latency of 2.85 ms and a bandwidth of 29 kbps. The integration with the TCP/IP model was facilitated through software written in Python, and Universal Software Radio Peripheral (USRP) software was used to define radio platforms with the GNU radio program for physical layer implementation. The experimental setup included a PLC link, a VLC downlink, and an IR uplink, showcasing the feasibility and performance of the hybrid communication system in indoor environments.The authors in [12] investigated the physical layer security of hybrid PLC and VLC networks, addressing a gap in the current research, which had previously largely focused on communication reliability and resource allocation. The study examined secure communication in a two-hop PLC/VLC network with two legitimate users employing non-orthogonal multiple access (NOMA), and considered the threats posed by eavesdroppers on both the PLC and VLC links. By analyzing the channel statistical characteristics influenced by the randomness of impulsive noise in the PLC link and user positions in the VLC link, the authors derived approximate closed-form expressions for secrecy outage probability (SOP). Their simulations validated the theoretical findings, highlighting the necessity of a joint parameter design for both links to achieve optimal secrecy performance. Their results demonstrated that the worst-case SOP scenario occurs when eavesdropping is possible on both the PLC and VLC links, compared to eavesdropping on a single link. The study in [10] presents a hybrid optical wireless network that integrates FSO and VLC to address the needs of space–air–ground–ocean communication architectures, particularly in RF-sensitive or security-requiring environments. The study defines the functional modules of the network coordinator and outlines the implementation and deployment details. Key network-layer mechanisms—user identification and localization, user mobility and handoff control, and routing and traffic management—are designed within the coordinator. An experimental platform evaluates the transmission performance of the hybrid network, demonstrating a complete data aggregation, transmission, and distribution procedure using heterogeneous interconnections. This includes a two-segment 1.0 m 450 Mb/s VLC transmission interconnected by a one-segment 430 m 0.96 Gb/s FSO transmission, maintaining a BER within the forward-error-correction limit. The study also experimentally assesses VLC performance at three speed levels and FSO performance under five air-quality conditions, confirming the feasibility and reliability of the proposed hybrid optical wireless network for high-data-rate, secure communication in various environments.

In terms of THz, similar to VLC, several studies have been conducted that combine it with either RF or FSO [13,14]. Some studies have been conducted on performance improvement that combine all three of them into one system; for example, in [6], the authors propose a hybrid FSO/RF-THz DF relay link. Here, an adaptive combining-based approach is used for the backhaul network by combining FSO and RF, and then, through the DF relay, the end user receives the information using a THz link. The authors derive expressions for the performance characteristics of this system, and then, through simulation results, state that this adaptive approach performs better compared to a single-link system. In [15], the authors investigate the potential of THz frequency bands for data transmissions between core networks and access points (AP) in next-generation wireless systems. They analyze a dual-hop THz-RF wireless system where an AP serves as a relay between the core network and the end-user equipment. The study employs a generalized end-to-end channel model with an independent and not identically distributed (i.ni.d.) fading for the THz and RF links, incorporating the α−μ distribution, pointing errors in the THz link, and an asymmetrical relay position. The authors derive a closed-form expression for the cumulative distribution function (CDF) of the E2E SNR, facilitating a generalized performance analysis over THz fading channels. Utilizing the derived CDF, they evaluate system performance using the DF protocol, deriving closed-form expressions for outage probability, SNR moments, average BER, and an the approximation of ergodic capacity. Additionally, they perform asymptotic analysis on the ergodic capacity, outage probability, and average BER in the high-SNR region, determining the system’s diversity order. The study also examines the system using an i.i.d. model to provide analytical insights under various practical scenarios. Through simulation and numerical analysis, the study reveals significant effects of THz link fading parameters and a nominal impact of normalized beam-width on the performance of the relay-assisted THz-RF system. In [16], the authors examined the performance of a mixed THz/FSO wireless transmission system, accounting for the joint effects of channel fading and pointing errors in both THz and FSO links. Utilizing a semi-blind amplify-and-forward (AF) protocol, they derived the exact expressions for outage probability, average BER, and average channel capacity. To provide further insights, asymptotic expressions for outage probability and average BER were also presented, revealing that the diversity gain is influenced by the channel fading and pointing errors of both links. The study extended to consider hardware impairments, deriving the outage probability for systems with non-ideal hardware, and further explored multi-antenna scenarios by obtaining asymptotic outage probability expressions. The numerical results illustrated that path loss, channel fading, pointing errors, and hardware impairments significantly degrade system performance, highlighting critical factors to address in the design and implementation of THz/FSO wireless systems. In [17], the authors investigate a hybrid FSO/THz-based backhaul network designed to deliver high-data-rate, reliable communication to terrestrial mobile users (MUs) operating in millimeter-wave (mmWave) bands. The FSO link faces challenges from atmospheric turbulence and pointing errors, while the THz link is impacted by high path loss, small-scale fading, and misalignment errors. To mitigate back-and-forth switching effects, a soft switching method is introduced at the AP to select signals from the hybrid FSO/THz link, and a performance comparison is conducted with hard switching methods. The study employs selective DF relaying at the AP, deriving closed-form expressions for individual link outage probability, E2E outage probability, asymptotic outage probability, ergodic capacity, and generalized average BER. The impact of parameters such as atmospheric turbulence, pointing/misalignment errors, link distance, atmospheric attenuation/path loss, the fading parameters of the THz and access links, and the number of antennas on network performance is analyzed. The results demonstrate that an appropriate switching method can significantly enhance the rate and reliability of backhaul links while maintaining a minimal switching overhead, thereby optimizing the performance of the integrated FSO/THz network.

Numerous studies have explored integrating VLC with FSO, RF, and PLC, and THz with FSO/RF, globally and in Portugal. However, regarding THz-VLC integration, as per the authors’ understanding, no studies have been conducted in Portugal, and only two have been published worldwide [18,19]. These two studies focused on enhancing communication networks for VR, addressing challenges like minimizing transmission delay and maximizing successful transmission probability. They did not compare THz-VLC performance with other communication technologies or evaluate system performance in terms of BER or outage probability.

This paper is structured to comprehensively address the proposed system. It starts with Section 1 outlining this study’s significance and objectives. Section 2 highlights this research’s driving factors and unique contributions. Section 3 discusses the collaborative benefits and obstacles of these systems. Practical applications are showcased in Section 4. Section 5 provides a detailed technical framework, followed by Section 6 evaluating system performance. Section 7 discuss the findings from the simulations. Section 8 proposes areas for further research. Finally, Section 9 summarizes our key findings.

## 2. Motivation and Contribution

Our literature review underscores the potential of cooperative relay assistance in enhancing overall communication system performance through interoperability among different communication technologies. While considerable research has been conducted on cooperative relay-assisted RF-VLC, RF-THz, VLC-FSO, and FSO-THz configurations, to the best of the authors’ knowledge at the time of writing, no studies have been published on the performance analysis of cooperative relay-assisted THz-VLC systems in terms of BER and outage probability.

Given the notable improvements in system performance achieved through the integration of THz and VLC technologies, this study endeavors to explore the potential benefits of a THz-VLC communication link facilitated by a DF relay architecture. The analysis focuses on BER, with the derivation of closed-form expressions for E2E metrics, marking the first attempt in the literature to do so.

The main contributions of this paper can be summarized as follows:

- The proposal of a dual-hop UAV-assisted communication system employing a DF relay architecture, utilizing THz communication in the primary hop and VLC in the secondary hop.
- The derivation of closed-form expressions for E2E BER with and without the presence of THz pointing errors.
- The presentation of simulation results to investigate the performance of the proposed system. A comparative analysis of BER and outage probability performances between the proposed system and FSO-VLC and RF-VLC configurations is conducted to highlight the advantages of integrating THz with VLC.

## 3. Synergy and Challenges of UAV-Assisted THz-VLC Systems

### 3.1. Synergy between THz and VLC in UAV Applications

The combination of THz and VLC in UAV applications provides a synergistic approach that harnesses the strengths of both communication methods. THz communication offers extremely high data rates due to the large available bandwidth [5]. This makes it ideal for transmitting large volumes of data quickly from the UAV to a relay point. The requirement for a clear line-of-sight (LoS) for THz communication is less of an issue for UAVs operating at high altitudes, where there are typically minimal obstructions [8]. On the other hand, VLC, which also requires an LoS, can leverage the vast and unregulated visible light spectrum to provide high-speed communication indoors. VLC can be seamlessly integrated into existing LED lighting systems, providing dual functionality of illumination and data transmission within buildings [4].

Moreover, UAVs can effectively utilize THz communication in outdoor environments, where they operate above many potential obstructions. This high-altitude advantage complements VLC’s indoor efficiency, where controlled lighting conditions mitigate issues such as multipath fading and interference. The environmental adaptability of this dual-hop system is a significant advantage, with THz providing robust outdoor communication capabilities and VLC ensuring reliable indoor connectivity.

### 3.2. Challenges in Integrating THz with UAV Platforms

**Figure 1:**
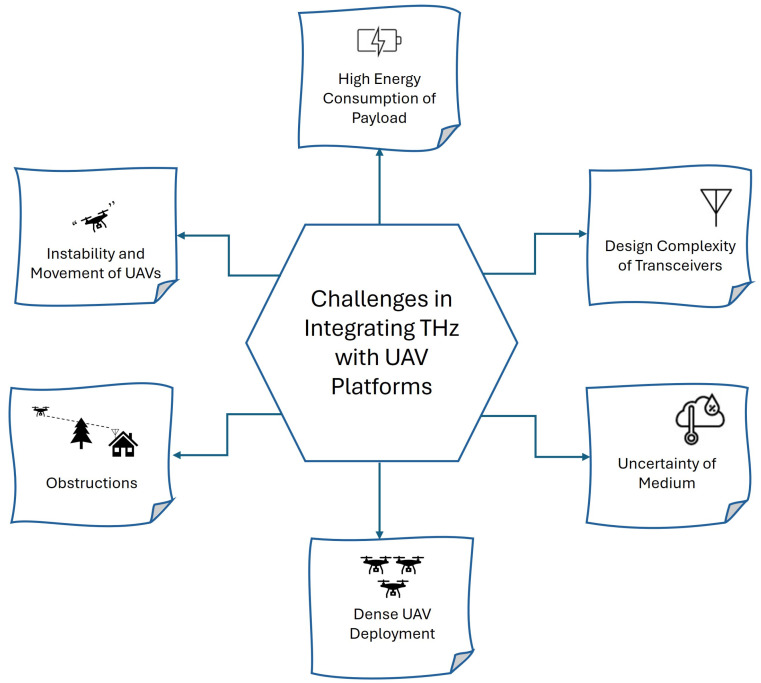
Challenges in integrating THz communication with UAV platforms. This diagram illustrates key challenges in implementing THz communication systems on UAV platforms, including high energy consumption of payload, design complexity of transceivers, uncertainty of medium, dense UAV deployment, obstructions, and instability and movement of UAVs.

The challenges associated with integrating THz with UAV platforms are illustrated in Figure 1 and are described below.

UAVs are prone to wobbling and unpredictable movements due to wind, turbulence, and rapid changes in direction. These factors can disrupt the THz signal transmission, affecting the reliability and stability of communication links [20].

THz communication systems and their associated payloads are power-intensive, requiring significant energy to operate. This high energy consumption reduces the UAV’s flight time and operational efficiency, necessitating advanced power management solutions [21].

Developing transceivers capable of operating efficiently at THz frequencies involves intricate design and engineering challenges. These transceivers must handle high data rates, maintain signal integrity, and operate within the limited size and weight constraints of UAVs [22].

THz signals are highly susceptible to blockages from physical objects such as buildings, trees, and even atmospheric particles. These obstructions can significantly degrade signal quality and limit the effective range and reliability of THz communication systems [22].

Deploying a large number of UAVs in a confined area can lead to interference and signal congestion. Managing the spectrum and ensuring seamless communication among multiple UAVs require sophisticated coordination and interference mitigation strategies [20].

The propagation characteristics of THz signals are influenced by environmental conditions such as humidity, temperature, and atmospheric composition. This variability introduces uncertainty in the communication medium, complicating the prediction and optimization of THz signal performance [21].

### 3.3. Challenges in Using VLC for Relay–Destination Links

**Figure 2:**
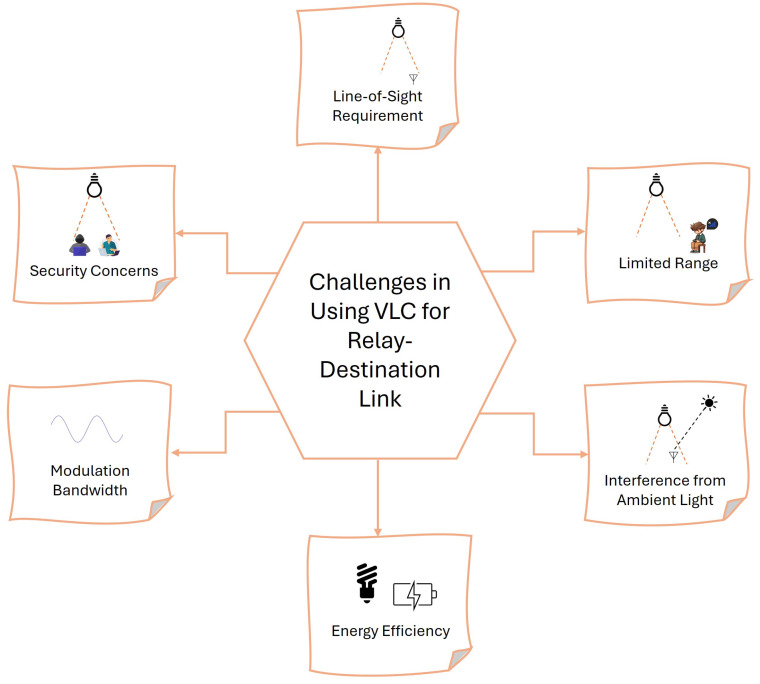
Challenges in using VLC for relay–destination links. This diagram highlights the primary challenges associated with employing VLC in relay–destination links, including LoS requirement, limited range, interference from ambient light, energy efficiency, and modulation bandwidth.

The challenges associated with using VLC for relay–destination links are illustrated in Figure 2 and are described below.

VLC relies heavily on a clear LoS between the transmitter and receiver. Obstacles such as furniture, walls, and even people can block the light path, leading to interruptions in the communication link [23].

The effective range of VLC systems is typically confined to the immediate vicinity of the light source. This limitation restricts the coverage area and necessitates multiple light sources for broader coverage, increasing complexity and cost [24].

Ambient light sources, such as sunlight or other artificial lighting, can cause interference with VLC signals. This interference can degrade signal quality and reduce communication reliability, especially in environments with varying lighting conditions [25].

Maintaining efficient energy consumption while ensuring adequate illumination and communication quality poses a challenge. Balancing the dual role of lighting and data transmission requires advanced control mechanisms to optimize power usage without compromising performance [4].

The modulation bandwidth of LEDs used in VLC is limited, restricting data transmission rates. Enhancing the modulation capabilities of LEDs to support higher data rates involves sophisticated engineering and can impact the cost and complexity of the system [23].

VLC systems are susceptible to eavesdropping and unauthorized access, as the communication medium (light) can easily be intercepted by unintended receivers within the coverage area. Implementing robust security measures to protect data transmission is essential to ensure privacy and prevent data breaches [25].

Addressing these challenges involves innovative approaches in materials science and communication technology design to create efficient, compact, and lightweight systems suitable for UAV deployment. Although we do not directly tackle these integration issues in this study, we provide a foundational framework and valuable insights that set the stage for future research to tackle these integration challenges, guiding the development of effective solutions.

## 4. Selected Use Cases and Opportunities

In this section, four selected use cases of UAV-assisted THz-VLC systems will be discussed.

### 4.1. Healthcare

**Figure 3:**
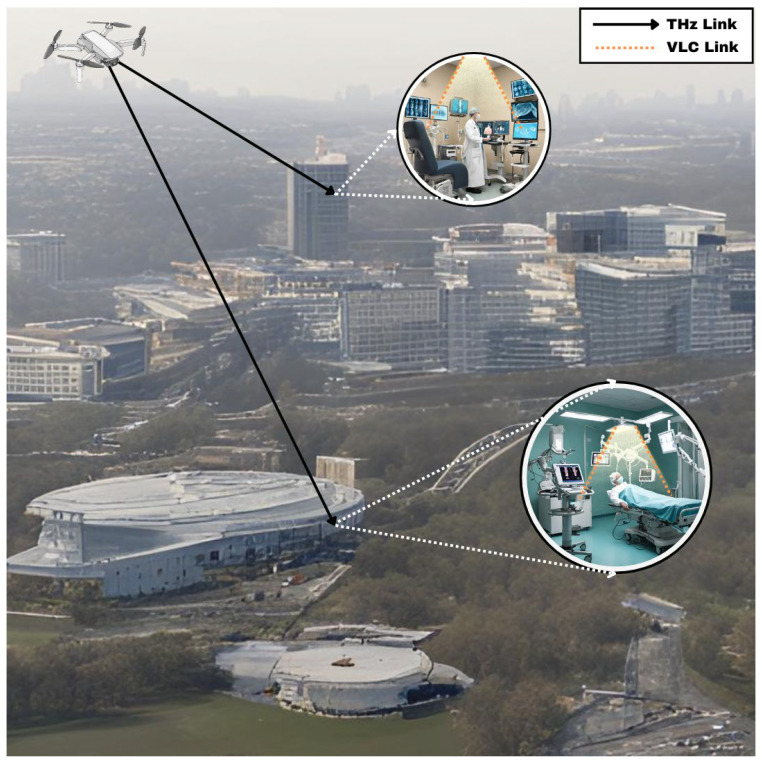
This diagram illustrates the use of THz and VLC in a healthcare setting. The system employs a UAV to facilitate dual-hop communication between a hospital and a remote clinic, enhancing data transfer capabilities for medical applications. THz communication is utilized for outdoor communication, while indoor communication utilizes VLC.

UAV-assisted THz-VLC systems can be utilized in a healthcare setting to transmit patient medical information to a nearby laboratory, as depicted in Figure 3. Patient medical information is transmitted from the sender using a VLC uplink to a DF relay within the hospital. The DF relay decodes the received information and retransmits it to a UAV using THz communication. At the UAV node, an AF relay architecture is used to enhance the SNR of the transmitted signal by amplifying and forwarding it to another DF relay positioned at the laboratory at a different location. The DF relay at the laboratory decodes the information and retransmits it to the lab technician using a downlink VLC. Utilizing VLC ensures that interference with sensitive equipment in the hospital and laboratory is avoided, while THz communication enables high-speed information transmission.

### 4.2. Underwater

**Figure 4:**
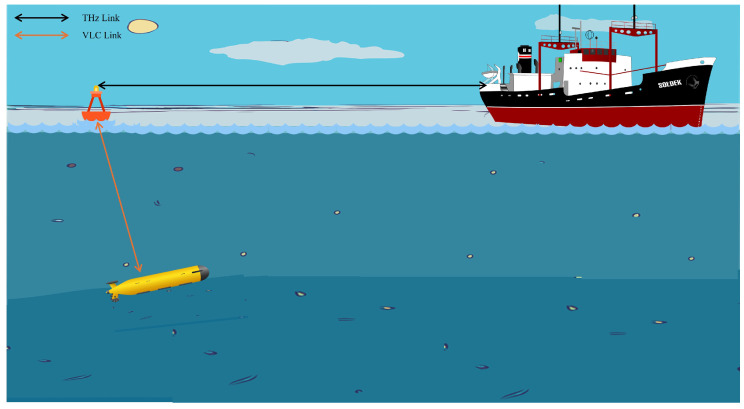
This diagram illustrates the use of THz and VLC in an underwater wireless communications application. The system employs a floating buoy to facilitate dual-hop communication between a ship and an AUV, enhancing data transfer capabilities for underwater applications. THz communication is utilized for ship-to-buoy communication, while laser-based VLC is utilized for underwater communication.

The information can be transmitted from the onshore base station to an Autonomous Underwater Vehicle (AUV) via a floating buoy equipped with DF relay architecture, as depicted in Figure 4. The signal from the onshore base station to the floating buoy will utilize THz communication, while the retransmission from the floating buoy to the AUV will utilize laser-based VLC. Lasers are preferred for deep-sea communication over LEDs due to their ability to extend the link distance. However, the use of lasers introduces pointing errors to the system due to the high turbulence experienced underwater. Appropriate mitigation techniques should be employed to maximize the system’s reliability in this setting.

### 4.3. Military

**Figure 5:**
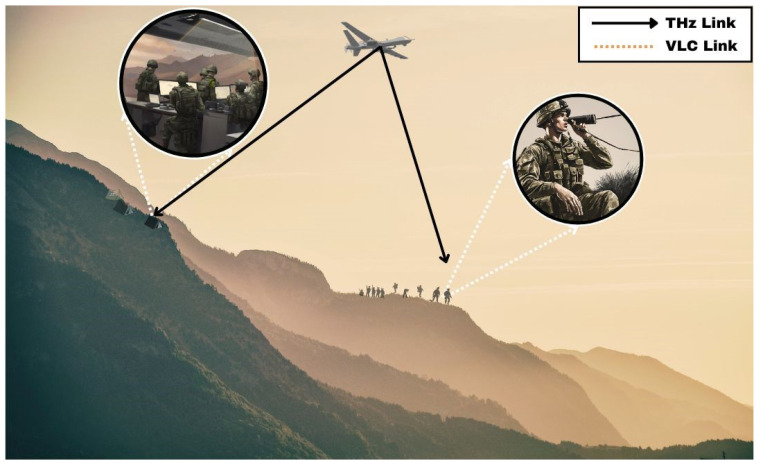
This diagram illustrates the use of THz and VLC in a military application. The system employs a fixed-wing UAV to facilitate dual-hop communication between a command center and the ground troops. THz communication is utilized for outdoor communication, while indoor communication utilizes VLC.

In this scenario, ground troops operating in a hostile environment could establish a secure communication link with the command centre using a relay station deployed in an intermediate location using a triple-hop DF and AF protocol-based THz-VLC system, as depicted in Figure 5. The first hop of the communication link would utilize THz uplink communication between the ground troops and the relay station. THz waves offer high bandwidth and can penetrate various obstacles encountered in the battlefield, allowing for reliable communication over short to medium distances. The relay station will amplify and forward the information to the relay at the command center again through THz communication. In the third and final hop, the relay at the command center will decode the information and retransmit it to the team inside the command center using a VLC downlink. The use of VLC ensures that the communication remains covert and immune to detection by adversaries.

### 4.4. Video Streaming

**Figure 6:**
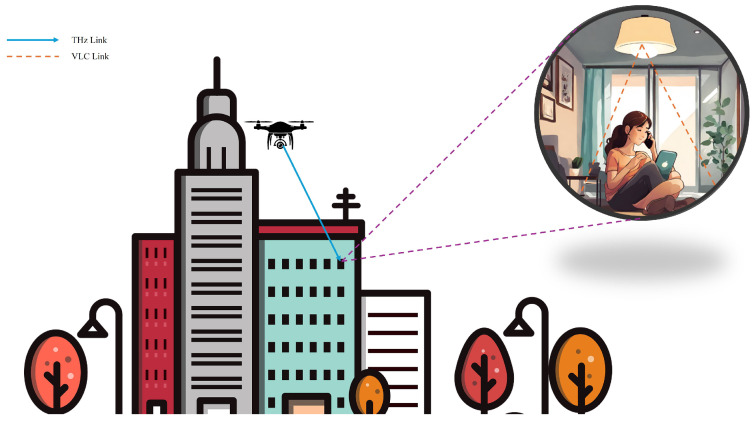
This diagram illustrates the use of THz and VLC in a 4K video streaming scenario. THz communication is utilized for outdoor communication from the UAV, while indoor communication utilizes VLC.

In this scenario, the UAV captures video footage and transmits it to a relay node using THz communication. THz frequencies are ideal for high-bandwidth applications like video streaming due to their ability to support high data rates, enabling the transmission of high-resolution video with minimal latency. The relay node, positioned to maintain a stable connection with the UAV, receives the video data and then forwards it to the end user inside the building using VLC, as illustrated in Figure 6.

Using VLC for the final leg of transmission inside the building is advantageous for several reasons. VLC can leverage existing lighting infrastructure, such as LED lights, to transmit data, reducing the need for additional hardware. Furthermore, VLC provides a secure communication channel as the light waves are confined within the room, minimizing the risk of eavesdropping from outside the building. This dual-mode communication approach, combining THz for long-range, high-bandwidth transmission and VLC for secure, indoor delivery, ensures efficient and reliable video streaming from the UAV to the end user.

A similar use case will be analyzed in detail in the next few sections.

## 5. System Model

**Figure 7:**
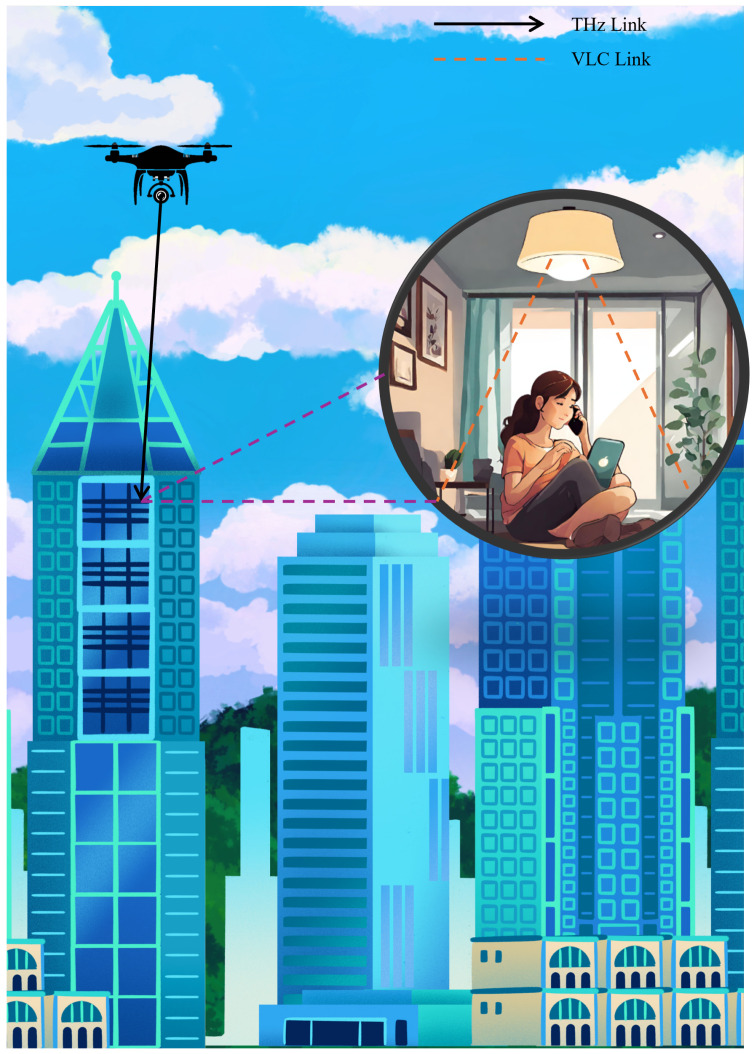
System model overview.

The system model comprises a dual-hop THz-VLC decode-and-forward relaying system. In this model, the first hop utilizes a THz channel to transmit information from the UAV (source node) to the relay node. The relay node decodes the received signal and retransmits it through VLC inside the building, as shown in Figure 7.

The system-level equations are described below:

(1) $$Y_{sr}=\sqrt{P_{s}}H_{THz}x+n_{SR}$$

(2) $$Y_{rd}=\sqrt{P_{r}}H_{VLC}\tilde{x}+n_{RD}$$

where $P_{s}$ represents the source power, $P_{r}$ denotes the relay power, $H_{THz}$ signifies the THz channel from source to relay, $H_{VLC}$ denotes the VLC channel from relay to destination, *x* symbolizes the information signal, $\tilde{x}$ stands for the signal encoded by the relay, and $n_{SR}$ and $n_{RD}$ represent the noise from source to relay and relay to destination, respectively [7].

### 5.1. THz Channel Model

The THz channel gain is represented by

(3) $$H_{THz}=h_{p}\cdot h_{a}\cdot h_{m},$$

where $h_{p}$, $h_{a}$, and $h_{m}$ denote the free-space path loss, molecular absorption loss, and misalignment fading effect, respectively.

#### 5.1.1. Free-Space Path Loss (FSPL)

The line-of-sight (LOS) free-space path loss can be expressed using the Friis equation [5]:

(4) $$h_{p}=\frac{c\sqrt{G_{TX}G_{RX}}}{4\pi df},$$

where *d* represents the distance between the transmitter and the receiver, *f* is the carrier frequency, and $G_{TX}$ and $G_{RX}$ signify the gain of the transmitter and receiver antenna, respectively.

#### 5.1.2. Molecular Absorption Loss

Molecular absorption loss refers to the attenuation of THz waves due to the absorption of specific molecules in the transmission window. Molecules such as $O_{2}$ and $H_{2}O_{g}$ absorb a portion of the energy emitted by THz signals, leading to divisions in the THz frequency spectrum [26]. When $k_{a}\left(f\right)$ represents the absorption coefficient, the absorption loss can be modeled as

(5) $$h_{a}=e^{-\frac{1}{2}k_{a}\left(f\right)d}.$$

#### 5.1.3. Misalignment Fading Effect

THz transceivers with directional antenna systems are susceptible to building sway and environmental factors, resulting in misaligned antenna beams and pointing errors. Considering a receiver with a circular aperture area of radius *a*, the transmitter emits a symmetric beam characterized by a waist $w_{d}$ at a certain distance *d*. Then the equivalent beam width is expressed as

(6) $$w_{eq}=\sqrt{w_{d}^{2}\frac{\sqrt{\pi }\mathrm{erf}\left(u\right)}{2u\exp(-u^{2})}},$$

where $u=\frac{\sqrt{\pi }a}{\sqrt{2}w_{d}}$. The misalignment gain is expressed as

(7) $$h_{m}\left(r\right)\approx A_{0}\exp\left(-\frac{2r^{2}}{w_{eq}^{2}}\right),$$

where $A_{0}$ represents the fraction of power collected by the receiver, expressed as $A_{0}={\left(erf\left(u\right)\right)}^{2}$. The Probability Density Function (PDF) of the misalignment fading effect is expressed as

(8) $$f_{h_{m}}\left(x\right)=\frac{\xi^{2}}{A_{0}^{\xi^{2}}}x^{\xi^{2}-1},0\leq x\leq A_{0},$$

where ξ denotes the ratio between the equivalent beam width and the standard deviation of the pointing error at the receiver, expressed as $\xi =\frac{w_{eq}}{2\sigma_{r}}$.

### 5.2. VLC Channel Model

In this study, it is assumed that the LOS path is the sole means of communication between the photodiode (PD) and the LED. The expression for the LoS direct current (DC) channel gain in VLC is

(9) $$\begin{array}{c} H_{VLC}=\left\{\begin{array}{cc} \frac{A_{pd}L_{0}\left(\theta \right)}{D^{2}}cos\left(\psi \right)T_{s}\left(\psi \right)g_{s}\left(\psi \right), & 0<\psi <\psi_{c}\\ 0, & \psi >\psi_{c}, \end{array}\right. \end{array}$$

where $A_{pd}$ denotes the area of the photodiode, $L_{0}$ represents the LED radiation distribution function, *D* is the distance between the transmitter and the receiver, $T_{s}\left(\psi \right)$ signifies the filter gain, $g_{s}\left(\psi \right)$ represents the concentrator gain, and ψ denotes the FoV of the photodiode.

The radiation distribution function, considering the number of radiation patterns, *m*, and the radiation angle of the LED, θ, can be expressed as

(10) $$L_{0}\left(\theta \right)=\frac{m+1}{2\pi }\cos^{m}\left(\theta \right).$$

## 6. Performance Analysis

If $P_{s}$ is the source power and $\sigma_{SR}$ is the noise power corresponding to $n_{SR}$, then the SNR for the THz link with the presence of pointing errors can be expressed as

(11) $$\gamma_{T}=\frac{P_{s}{\left|H_{THz}\right|}^{2}}{\sigma_{SR}^{2}}.$$

Considering a scenario with no pointing errors in the THz link, the SNR of the THz link can be expressed as

(12) $$\gamma_{T_{NP}}=\frac{P_{s}{\left|h_{p}h_{a}\right|}^{2}}{\sigma_{SR}^{2}}.$$

If $P_{r}$ is the relay power and $\sigma_{RD}$ is the noise power, then the SNR for the VLC link can be expressed as

(13) $$\gamma_{VLC}=\frac{P_{r}{\left|H_{VLC}\right|}^{2}}{\sigma_{RD}^{2}}$$

**Lemma** **1.** *Consider a dual-hop decode-and-forward relay-assisted THz-VLC link. Assuming the presence of pointing errors in the THz link and deterministic fading in the VLC link, and utilizing Binary Phase-Shift Keying (BPSK) modulation for the THz link along with on–off keying (OOK) modulation for the VLC link, the E2E BER can be expressed as in Equation (14):*

(14) $$\begin{array}{cc} BER_{E2E}= & \frac{\xi^{2}}{4{\left(\nu A_{0}^{2}{\left|\sqrt{P_{s}}h_{p}h_{a}\right|}^{2}\right)}^{\frac{\xi^{2}}{2}}}\Gamma \left(\nu A_{0}^{2}{\left|\sqrt{P_{s}}h_{p}h_{a}\right|}^{2},\frac{\xi^{2}}{2}\right)\\ & +\frac{1}{2}\mathrm{erfc}\left(\sqrt{\frac{\gamma_{VLC}}{2}}\right)-2\left(\frac{\xi^{2}}{4{\left(\nu A_{0}^{2}{\left|\sqrt{P_{s}}h_{p}h_{a}\right|}^{2}\right)}^{\frac{\xi^{2}}{2}}}\Gamma \left(\nu A_{0}^{2}{\left|\sqrt{P_{s}}h_{p}h_{a}\right|}^{2},\frac{\xi^{2}}{2}\right)\cdot \frac{1}{2}\mathrm{erfc}\left(\sqrt{\frac{\gamma_{VLC}}{2}}\right)\right). \end{array}$$

**Proof.**  Given that misalignment fading stands as the sole random fading component, assuming ν represents the average SNR of an uncoded communication system, as referenced in [5], the instantaneous BER of the THz link can be expressed as:

(15) $$BER_{THz}=E_{f_{h_{m}}}\left[Q\left(\sqrt{2|\sqrt{P_{s}}h_{p}h_{a}{|}^{2}{\left|h_{m}\right|}^{2}\bar{\nu }}\right)\right]$$

Here, *Q*(.) denotes the tail distribution function of the Gaussian distribution, and $E_{f_{h_{m}}}$ [.] signifies the expected value of the misalignment PDF. Consequently, the BER expression can be formulated as

(16) $$BER_{THz}=\frac{\xi^{2}}{A_{0}^{\xi^{2}}}\int_{0}^{A_{0}}Q\left(\sqrt{2\bar{\nu }{\left|\sqrt{P_{s}}h_{p}h_{a}\right|}^{2}x^{2}}\right)x^{\xi^{2}-1}dx$$

Drawing parallels with [5], this study employs the Chernoff exponential bound to approximate the Gaussian *Q* function, expressed as $Q\left(x\right)\leq \frac{1}{2}\exp\left(-\frac{1}{2}x^{2}\right),x>0$. Incorporating this approximation, the BER expression can be reformulated as

(17) $$BER_{THz}=\frac{\xi^{2}}{4{\left(\nu A_{0}^{2}{\left|h_{p}h_{a}\right|}^{2}\right)}^{\frac{\xi^{2}}{2}}}\Gamma \left(\nu A_{0}^{2}{\left|h_{p}h_{a}\right|}^{2},\frac{\xi^{2}}{2}\right)$$

where Γ(.,.) is the lower incomplete gamma function. Given that the VLC link uses a deterministic path loss model, the BER can be expressed as

(18) $$BER_{VLC}=\frac{1}{2}erfc\sqrt{\frac{\gamma_{VLC}}{2}}$$

The E2E BER for the THz-VLC link can be obtained through the unified error rate expression in [27] as follows:

(19) $$\begin{array}{cc} BER_{THz-VLC}= & BER_{THz}+BER_{VLC}-2\cdot BER_{THz}\cdot BER_{VLC}. \end{array}$$

Substituting Equations (17) and (18) into (19) proves (14).

This way, the E2E BER for a dual-hop decode-and-forward relay-assisted THz-VLC link with pointing errors in the THz link and deterministic fading in the VLC link, using BPSK and OOK modulation, is derived using the expected value of the misalignment PDF and the Chernoff exponential bound approximation for the Gaussian Q function. □

**Lemma** **2.** *Consider a dual-hop decode-and-forward relay-assisted THz-VLC link. Assuming deterministic fading in the entire THz-VLC link, and utilizing BPSK modulation for the THz link along with OOK modulation for the VLC link, the E2E BER can be expressed as*

(20) $$\begin{array}{cc} BER_{E2E}= & Q\left(\sqrt{2\gamma_{T_{NP}}}\right)+\frac{1}{2}erfc\sqrt{\frac{\gamma_{VLC}}{2}}-2\left(Q\left(\sqrt{2\gamma_{T_{NP}}}\right)\cdot \frac{1}{2}erfc\sqrt{\frac{\gamma_{VLC}}{2}}\right). \end{array}$$

**Proof.**  The instantaneous BER of the THz link considering deterministic fading using BPSK modulation can be expressed as [28],

(21) $$BER_{BPSK}=Q\left(\sqrt{2\gamma_{inst}}\right),$$

where $\gamma_{inst}$ is the instantaneous SNR. Substituting (12) into (21) gives

(22) $$BER_{THz}=Q\left(\sqrt{2\gamma_{T_{NP}}}\right).$$

Substituting (18) and (22) into (19) gives (20).

This way, the E2E BER for a dual-hop decode-and-forward relay-assisted THz-VLC link with deterministic fading in both links, using BPSK modulation for the THz link and OOK modulation for the VLC link, is derived using the Q function for the instantaneous SNR and error function for the VLC link.

In the context of the system model, outage occurs when either the SNR of the THz link or the VLC link falls below or equals a predefined threshold, denoted as $\gamma_{\mathrm{th}}$, which can be derived as follows:

(23) $$\begin{array}{c} P_{\mathrm{outage}}=P\left(\gamma_{DF}\leq \gamma_{th}\right)=P\left[min\left(\gamma_{T},\gamma_{VLC}\leq \gamma_{th}\right)\right]. \end{array}$$

Consequently, the independence of the instantaneous SNR for each of link is defined in Equations (11) and (13), which can be further expanded and rewritten as

(24) $$\begin{array}{cc} P_{\mathrm{outage}} & =P\left(|H_{THz}{|}^{2}\leq \frac{\gamma_{\mathrm{th}}}{P_{s}}\sigma_{SR}^{2}\right)+P\left(|H_{VLC}{|}^{2}\leq \frac{\gamma_{\mathrm{th}}}{P_{r}}\sigma_{RD}^{2}\right)\\ & -P\left(|H_{THz}{|}^{2}\leq \frac{\gamma_{\mathrm{th}}}{P_{s}}\sigma_{SR}^{2}\right)\cdot P\left(|H_{VLC}{|}^{2}\leq \frac{\gamma_{\mathrm{th}}}{P_{r}}\sigma_{RD}^{2}\right). \end{array}$$

□

## 7. Simulation Results

In this section, MATLAB was used for the simulations to validate the analysis and assess the performance of the cooperative relay-assisted THz-VLC link in terms of BER and outage probability.

All the parameters used for the simulation are listed in Table 1, they were selected based on credible literature sources.

**Figure 8:**
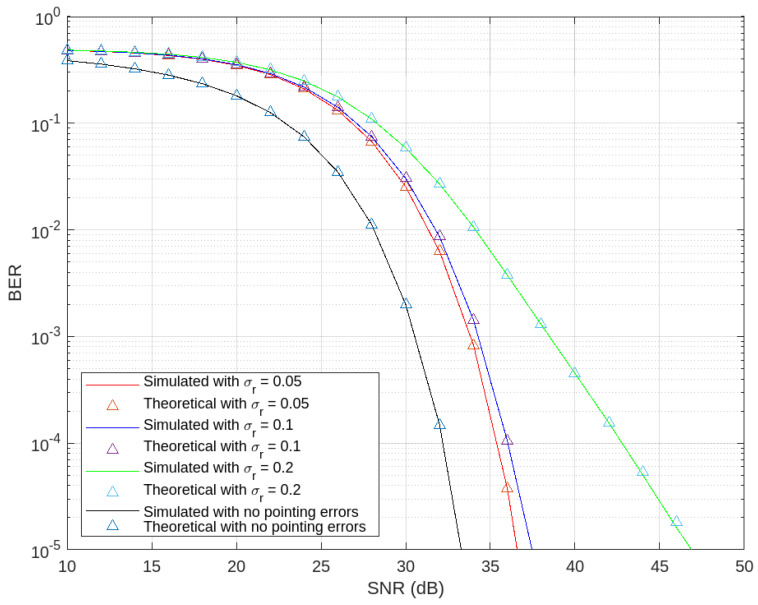
Comparison of BER performance for varying pointing errors in THz–VLC link.

Figure 8 illustrates the influence of pointing errors on the BER performance of the proposed THz-VLC scheme. An increase in BER corresponding to heightened pointing errors highlights the system’s sensitivity to alignment inaccuracies. The system demonstrates optimal performance under ideal conditions without pointing errors, underscoring the critical need for precise alignment. Additionally, the close match between the simulated BER and theoretical BER in the absence of pointing errors validates the theoretical framework presented in Lemma 2. This correlation confirms the robustness and accuracy of the theoretical model in predicting system performance under ideal conditions. Theoretical and simulation models from the literature [5] confirm that lower jitter variance is necessary for a longer transmission distance. Considering this and the values provided in Table 1, for the remainder of the simulations in this study, a jitter variance of 0.05 m^2^ is utilized.

**Figure 9:**
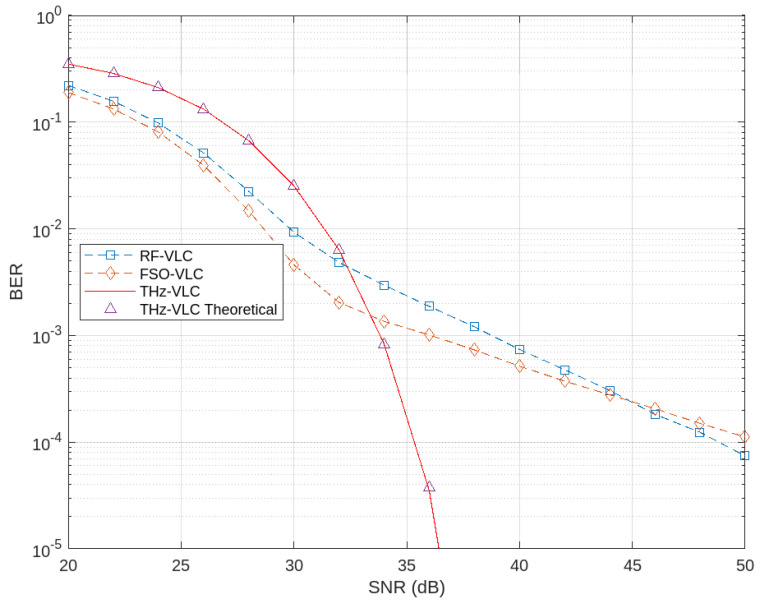
Comparison of BER performance among THz–VLC, RF–VLC, and FSO–VLC.

In Figure 9, the BER performance is compared across three different communication paradigms: RF-VLC, FSO-VLC, and THz-VLC. The first link from the source to the relay (using RF, THz, or FSO) employs BPSK modulation with a link distance of 30 m, while the subsequent VLC link uses OOK with a link distance of 5 m. This figure illustrates that RF-VLC and FSO-VLC outperform THz-VLC under lower SNR conditions. This is mainly due to the shorter wavelengths of THz signals compared to RF and optical signals, making them more susceptible to absorption by atmospheric gases and attenuation from obstacles, resulting in higher signal loss and degradation at lower SNR levels. However, as the SNR increases, THz-VLC exhibits superior performance, achieving a BER of $10^{-5}$ at a significantly lower SNR compared to FSO-VLC and RF-VLC. The disadvantages of the THz link, such as higher absorption and attenuation, diminish with increasing SNR. Additionally, THz signals offer higher bandwidth and data rates compared to RF and optical signals, allowing THz-VLC to leverage this advantage more effectively with higher SNR, leading to improved BER performance.

Furthermore, in Figure 9, the simulated BER for the THz-VLC link aligns closely with the theoretical BER, providing further validation for Lemma 1.

**Figure 10:**
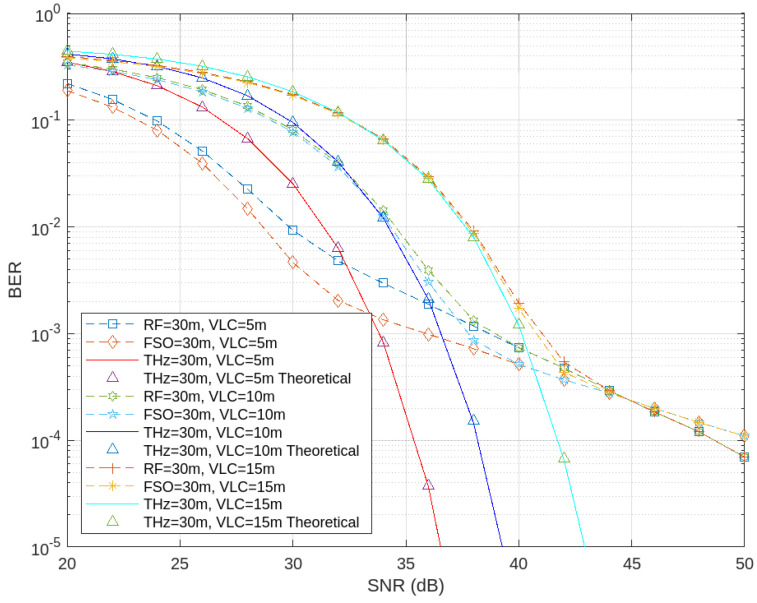
Comparison of BER performance among THz–VLC, RF–VLC, and FSO–VLC with varying relay–destination link distances.

An assessment was conducted by varying the distances for the second hop over VLC while maintaining a constant distance for the first hop (THz, RF, FSO). The results are illustrated in Figure 10.

As the relay–destination distance increases, the BER correspondingly rises due to higher attenuation and other losses over longer distances. An interesting observation emerges with increasing relay–destination link distance: the BER discrepancy between THz-VLC and the other systems notably diminishes under lower SNR conditions. This indicates that at extended distances, even under lower SNR conditions, THz-VLC has the potential to outperform RF-VLC and FSO-VLC. Furthermore, as shown in Figure 9, THz-VLC maintains its superiority over FSO-VLC and RF-VLC at higher SNR levels. This is because THz-VLC demonstrates resilience against the challenges posed by longer distances, even under lower SNR conditions, thereby narrowing the performance gap between THz-VLC and other systems.

These results suggest that in scenarios where the VLC link distance is greater, incorporating THz into the system yields superior performance, even under lower SNR conditions, compared to integrating FSO or RF.

**Figure 11:**
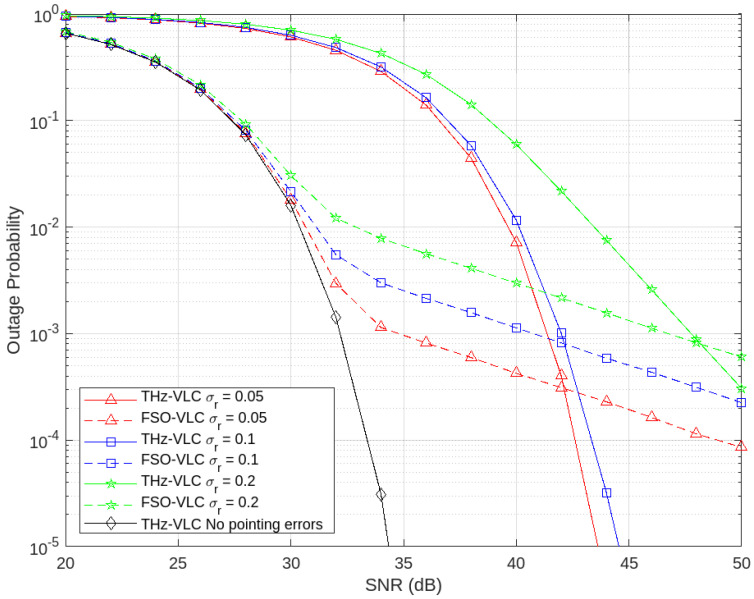
Outage probability performance comparison with varying pointing errors in THz–VLC and FSO–VLC.

Figure 11 compares the outage performance of the proposed THz-VLC system with the FSO-VLC system under varying pointing errors. Since RF does not experience pointing errors, the RF-VLC configuration is not included. Similar to the BER comparison, FSO-VLC performs better at lower SNRs, but the proposed THz-VLC system surpasses it at higher SNRs. This figure further highlights pointing error as a significant factor influencing system performance, with a lower pointing error contributing to improved system efficiency. Specifically, the system exhibits superior outage performance under the assumption of no pointing error. This indicates that maintaining precise alignment in the THz-VLC link is crucial for minimizing the likelihood of outages and ensuring reliable communication. A potential solution to this could be to dynamically optimize the position of the UAV to minimize pointing errors.

**Figure 12:**
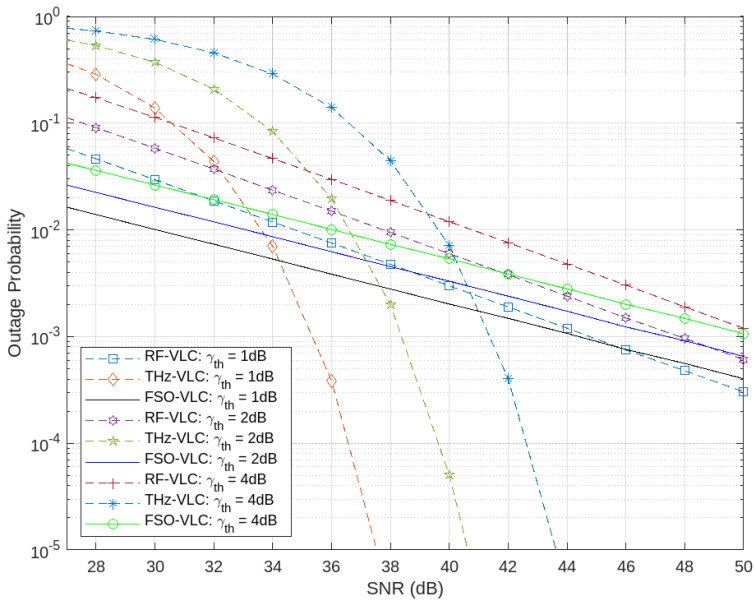
The outage probability performance comparison with varying $\gamma_{th}$ for THz–VLC, RF–VLC, and FSO–VLC.

Figure 12 illustrates the comparison of outage probability for the proposed system (THz-VLC) with FSO-VLC and RF-VLC across varying threshold SNRs ($\gamma_{th}$). A clear trend emerges wherein the outage probability increases with rising threshold SNRs for all configurations. This is expected because higher threshold SNRs set more stringent requirements for the minimum acceptable signal quality, making it harder to meet these criteria and thus increasing the likelihood of an outage.

Notably, despite having the highest threshold SNR, the THz-VLC system exhibits superior performance compared to FSO-VLC and RF-VLC, even when those systems are operating at their lowest threshold SNRs. This suggests that the THz-VLC system is more resilient and capable of maintaining lower outage probabilities even under more challenging conditions. The superior performance of the THz-VLC system can be attributed to its higher bandwidth and data rate capabilities, which allow it to handle higher SNR thresholds more effectively than RF and FSO systems.

## 8. Future Directions

**Figure 13:**
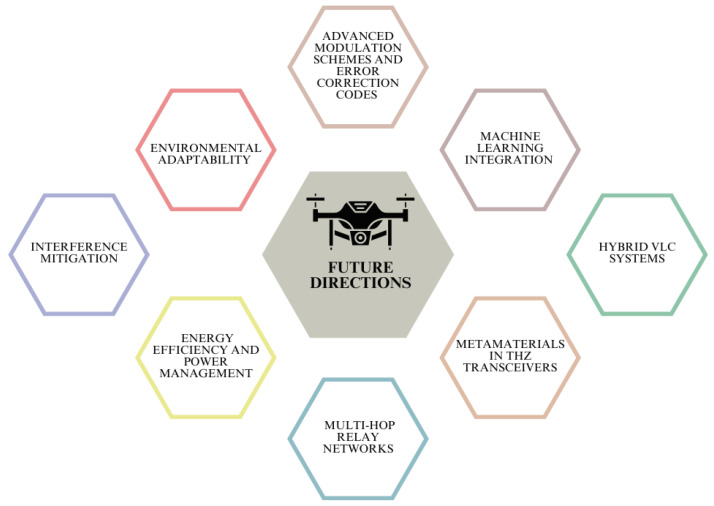
Illustration of future directions in THz-VLC systems.

Future research could explore several improvements and new technologies to enhance the performance and versatility of the THz-VLC system, as illustrated in Figure 13.

### 8.1. Advanced Modulation Schemes and Error Correction Codes

Using advanced modulation schemes like Quadrature Amplitude Modulation (QAM) can boost data rates and make THz-VLC systems more efficient. Employing Low-Density Parity-Check (LDPC) codes or Turbo codes can significantly reduce the bit error rate (BER). For example, LDPC codes excel at correcting errors in high-data-rate communications, which is crucial for maintaining data integrity in THz-VLC links.

### 8.2. Machine Learning Integration

Machine learning algorithms, such as Reinforcement Learning (RL), can optimize adaptive beamforming and dynamic channel estimation. These algorithms learn from the environment, adjusting the UAV’s position and orientation for optimal signal transmission and reception. A neural network-based channel estimator can predict and compensate for channel variations in real time, improving the communication link’s robustness against environmental changes and pointing errors.

### 8.3. Hybrid VLC Systems

Combining LEDs and lasers in a hybrid VLC system can harness the broad coverage of LEDs and the high data rates of lasers. LEDs can provide general illumination and broad coverage, while lasers are used for high-speed data transmission over longer distances. A system where LEDs handle ambient lighting and data broadcasting, while laser diodes are used for point-to-point high-speed communication within the same environment, optimizes overall system performance.

### 8.4. Metamaterials in THz Transceivers

Using metamaterials to design high-gain, compact THz antennas can enhance signal propagation and reduce losses. Metamaterials can be engineered to focus and direct THz waves more efficiently than conventional materials. Developing a THz antenna with a metamaterial lens that focuses the THz beam can reduce diffraction and improve the signal-to-noise ratio (SNR) for long-distance communication.

### 8.5. Multi-Hop Relay Networks

Implementing multi-hop relay networks where multiple UAVs act as intermediate relays extends the communication range and improves coverage. Each UAV decodes and forwards the signal to the next UAV or ground station. A cooperative communication strategy where UAVs use decode-and-forward (DF) relays to enhance signal quality and reliability over long distances can ensure robust communication even in challenging environments.

### 8.6. Energy Efficiency and Power Management

Developing energy-efficient hardware components and power management algorithms can extend UAV flight times. This includes using low-power electronics and optimizing flight paths to minimize energy consumption. Implementing a solar-powered UAV system that harnesses solar energy to power the communication payload and extend operational time can reduce reliance on battery power alone.

### 8.7. Interference Mitigation

Employing spectrum management techniques such as Dynamic Spectrum Access (DSA) can mitigate interference and optimize the use of available frequencies. DSA allows the system to dynamically switch to less congested frequencies. Using cognitive radio technology that senses the spectrum environment and adapts the frequency and transmission parameters can avoid interference with other communication systems.

### 8.8. Environmental Adaptability

Incorporating adaptive mechanisms that adjust to varying environmental conditions such as humidity and temperature can enhance the reliability of THz-VLC communication systems. These mechanisms include the real-time monitoring and adjustment of transmission power and beam direction. Developing an adaptive THz communication system that uses environmental sensors to monitor atmospheric conditions and dynamically adjust the transmission parameters can maintain optimal signal quality and reliability.

## 9. Conclusions

This paper introduced a dual-hop decode-and-forward relay-assisted communication system that combines terahertz and VLC. In this system, the first hop utilizes a THz channel to transmit information from the UAV (source node) to the relay node. The relay node decodes the received signal and retransmits it through VLC inside the building. This work presented the derivation of closed-form expressions for E2E BER with and without the presence of pointing errors, which were validated through simulations results.

Moreover, this study also conducted a comparative analysis of the proposed system with FSO-VLC and RF-VLC in terms of BER and outage probability. The results indicated the superior performance of THz-VLC compared to RF-VLC and FSO-VLC. Varying the VLC link distance while maintaining a constant distance between the source and relay indicated that integrating VLC with THz offers more system stability compared to FSO-VLC and RF-VLC due to the resilient nature of THz at higher SNRs.

These findings underscore the importance of considering factors such as path loss, pointing errors, and molecular absorption when deploying UAV-assisted THz-VLC system. Such considerations are paramount for optimizing performance and ensuring reliable communication in practical deployment scenarios.

## Figures and Tables

**Table 1:** Simulation parameters.

Parameters	Values
Number of bits	$1\times 10^{5}$
Distance [THz link]	30 m
Frequency [THz link]	0.3 THz
Radius of the reception antenna	0.1 m
Transmission beam footprint radius	0.6 m
Jitter variance, $\sigma_{r}$	0.05–0.2 m^2^
Distance [VLC link]	5 m
Photodiode area	0.05
Source power, $P_{s}$	1 dB
Relay power, $P_{r}$	1 dB
θ	53.13°
$\psi_{c}$	75°
ψ	53.13°
Refractive index of a lens	1.5
Distance [FSO link]	30 m
α [FSO link]	4.1
β [FSO link]	1.4
Distance [RF link]	30 m

## Data Availability

Data are contained within the article.
